# Temporal trends of molecular markers associated with artemether-lumefantrine tolerance/resistance in Bagamoyo district, Tanzania

**DOI:** 10.1186/1475-2875-12-103

**Published:** 2013-03-18

**Authors:** Maja Malmberg, Billy Ngasala, Pedro E Ferreira, Erik Larsson, Irina Jovel, Angelica Hjalmarsson, Max Petzold, Zul Premji, José P Gil, Anders Björkman, Andreas Mårtensson

**Affiliations:** 1 Malaria Research, Department of Medicine Solna, Karolinska Institutet, Stockholm, Sweden; 2 Department of Parasitology, Muhimbili University of Health and Allied Sciences, Dar-es-Salaam, Tanzania; 3 Centre of Molecular and Structural Biomedicine, Institute of Biotechnology and Bioengineering, University of Algarve, Faro, Portugal; 4 Departamento de Parasitología, Escuela de Microbiología, Facultad de Ciencias Universidad Nacional Autónoma de Honduras (UNAH), Tegucigalpa, Honduras; 5 Centre for Applied Biostatistics, Sahlgrenska Academy, University of Gothenburg, Gothenburg, Sweden; 6 Drug resistance Unit, Division of Pharmacogenetics, Department of Physiology and Pharmacology, Karolinska Institutet, Stockholm, Sweden; 7 Department of Biological Sciences, The Harpur College of Arts and Sciences, Binghamton University, Binghamton, NY, USA; 8 Division of Global Health (IHCAR), Department of Public Health Sciences, Karolinska Institutet, Stockholm, Sweden

**Keywords:** *Plasmodium falciparum*, Malaria, *pfmdr1*, *pfcrt*, Artemether-lumefantrine, Lumefantrine, Drug resistance, Tanzania

## Abstract

**Background:**

Development and spread of *Plasmodium falciparum* resistance to artemisinin-based combination therapy (ACT) constitutes a major threat to recent global malaria control achievements. Surveillance of molecular markers could act as an early warning system of ACT-resistance before clinical treatment failures are apparent. The aim of this study was to analyse temporal trends of established genotypes associated with artemether-lumefantrine tolerance/resistance before and after its deployment as first-line treatment for uncomplicated malaria in Tanzania 2006.

**Methods:**

Single nucleotide polymorphisms in the *P. falciparum multidrug resistance gene 1* (*pfmdr1*) N86Y, Y184F, D1246Y and *P. falciparum chloroquine transporter gene* (*pfcrt*) K76T were analysed from dried blood spots collected during six consecutive studies from children with uncomplicated *P. falciparum* malaria in Fukayosi village, Bagamoyo District, Tanzania, between 2004–2011.

**Results:**

There was a statistically significant yearly increase of *pfmdr1* N86, 184F, D1246 and *pfcrt* K76 between 2006–2011 from 14% to 61% (yearly OR = 1.38 [95% CI 1.25-1.52] p < 0.0001), 14% to 35% (OR = 1.17 [95% CI 1.07-1.30] p = 0.001), 54% to 85% (OR = 1.21 [95% CI 1.03-1.42] p = 0.016) and 49% to 85% (OR = 1.33 [95% CI 1.17-1.51] p < 0.0001), respectively. Unlike for the *pfmdr1* SNP, a significant increase of *pfcrt* K76 was observed already between 2004–2006, from 26% to 49% (OR = 1.68 [95% CI 1.17-2.40] p = 0.005). From 2006 to 2011 the *pfmdr1* NFD haplotype increased from 10% to 37% (OR = 1.25 [95% CI 1.12-1.39] p < 0.0001), whereas the YYY haplotype decreased from 31% to 6% (OR = 0.73 [95% CI 0.56-0.98] p = 0.018). All 390 successfully analysed samples had one copy of the *pfmdr1* gene.

**Conclusion:**

The temporal selection of molecular markers associated with artemether-lumefantrine tolerance/resistance may represent an early warning sign of impaired future drug efficacy. This calls for stringent surveillance of artemether-lumefantrine efficacy in Tanzania and emphasizes the importance of molecular surveillance as a complement to standard *in vivo* trials.

## Background

Modern malaria control relies primarily on sustained efficacy of artemisinin-based combination therapy (ACT). Reports of artemisinin tolerant/resistant *Plasmodium falciparum* from Southeast Asia [1-3] constitute, therefore, a major threat to recent global malaria control achievements. Within the African continent there has to date been no clear evidence of artemisinin resistance, but an increased parasite positivity rate on day 1 after initiation of ACT treatment has been reported from Kenya [4].

ACT is a combination of an artemisinin derivative, which rapidly reduces the parasite load, and a long-acting partner drug that kills the remaining parasites and suggestively protects the artemisinin component from resistance development. During clearance of the long-acting partner drug re-infecting parasites are exposed to slowly declining drug concentrations during several weeks. This phenomenon, which is of particular importance in high transmission areas, may be the starting point for development of tolerance/resistance towards the long-acting partner drug. This could initially result in a shorter post-treatment prophylactic effect and eventually reduce the ACT to an artemisinin derivative monotherapy.

The most commonly used ACT in Africa is artemether-lumefantrine. It has been deployed as first-line treatment for uncomplicated malaria in Tanzania since 2006. Artemether-lumefantrine has shown to be highly efficacious with PCR-corrected cure rates exceeding 95% [5-7]. However, artemether-lumefantrine has been associated with selection of single nucleotide polymorphisms (SNPs) in genes associated with anti-malarial drug resistance among re-infections, as compared with baseline parasite characteristics [8-10]. The main SNPs are *P. falciparum multidrug resistance gene 1* (*pfmdr1*) N86, 184F and D1246 [8,9] and the *P. falciparum chloroquine transporter gene* (*pfcrt*) K76 allele [10]. This highlights the need of close surveillance of molecular markers as an early warning system of development and spread of anti-malarial drug resistance and an important tool for insights into drug resistance development.

The aim of this study was to assess temporal trends of molecular markers associated with anti-malarial drug resistance in a rural Tanzanian village before and after wide scale deployment of artemether-lumefantrine as first-line treatment of uncomplicated *P. falciparum* malaria.

## Methods

### Study area

The studies were conducted in Fukayosi village, Bagamoyo District, Coast region, Tanzania. Fukayosi dispensary serves a population of approximately 7,000 people. The catchment area is primarily rural. Malaria transmission is high and occurs throughout the year with peaks related to the rainy seasons in May to July (long rains) and December to January (short rains). *Plasmodium falciparum* is the predominant malaria species and *Anopheles gambiae* complex the main vector. In 2004, sulphadoxine-pyrimethamine was first-line treatment and amodiaquine second-line treatment for uncomplicated malaria. Since November 2006 artemether-lumefantrine has been the first-line treatment.

### Study design and population

Study 1 was a two-arm (artemether-lumefantrine *versus* sulphadoxine-pyrimethamine) efficacy trial conducted in 2004 [6]. Study 2 was an artemether-lumefantrine pharmacokinetic and pharmacodynamics study performed in 2006 [11]. Study 3 was a two-arm (efficacy *versus* effectiveness) artemether-lumefantrine clinical trial conducted in 2007 [7]. Study 4, Study 5 and Study 6 represent pretreatment blood samplings done in 2008, 2010 and 2011, respectively (unpublished data). In total, 777 patients were included in the present analysis.

The study population consisted of children ≤10 years with symptomatic, uncomplicated, microscopy confirmed *P. falciparum* infection. Detailed inclusion and exclusion criteria for Studies 1 to 3 have been reported elsewhere [5,7,11]. A brief descriptive summary of study population is presented in Table 1.

**Table 1:** Base line characteristics of the study population

	**Study 1**	**Study 2**	**Study 3**	**Study 4**	**Study 5**	**Study 6**
**Year**	2004	2006	2007	2008	2010	2011
**Study period**	May	Jun	May – Nov	Aug-Oct	Oct-Dec + Jan 2011	Apr-Jun
**Patients included (n)**	106	50	258	200	33	130
**Sex, female patients (%)**	51	62	54	49	49	50
**Age, months, median (range)**	31 (6–135)	48 (12–119)	31 (4–60)	36 (2–124)	N.D	60 (12–120)
**Weight, kg, median (range)**	12 (6–33)	13 (8–30)	12 (5–21)	12 (5–33)	13 (6–29)	15 (8–32)
**Parasites/μL, geometric mean (range)**	21,595 (2,000-160,000)	36,885 (2,120-200,400)	43,259 (2,700-192,320)	17,531 (2,000-192,000)	18,537 (2,400-185,120)	10,198 (1,080-112,800)
**Slide positivity rate, microscopy positive/screened (%)**	175/434 (40%)	148/277 (53%)	718/1403 (51%)	473/745 (63%)	99/588 (17%)	599/1002 (60%)

### Biological material

Blood spots were collected on filter paper (Whatman 3 MM) just prior to initiation of anti-malarial treatment, dried and put in individual zipper plastic bags. Thereafter, they were transported to Karolinska Institutet, Sweden, for molecular analysis.

### Molecular analysis

#### DNA extraction and analysis

Genomic DNA was extracted from the dried blood spots using the BloodPrep™ Chemistry on an ABI PRISM^®^ 6100 (Applied Biosystems™, Fresno, CA, USA) according to the manufacturer’s instructions, and analysed by PCR for the presence of different genetic markers associated with anti-malarial drug resistance.

### Genotyping of *pfmdr1* and *pfcrt*

*Pfmdr1* N86Y and *pfcrt* K76T were genotyped using PCR-RFLP according to previously described protocol [12]. Restriction fragments were loaded on 2% agarose gels containing 0.1 μg/ml ethidium bromide, separated by electrophoresis and visualized under UV transillumination (GelDoc System, Biorad, Hercules, CA, USA). *Pfmdr1* Y184F was analysed by pyrosequencing in Study 1 [13], sequencing in Studies 2 and 3 [14] and PCR-RFLP in Studies 4, 5 and 6. *Pfmdr1* D1246Y was analysed by PCR-RFLP in Studies 1, 4, 5 and 6 [13], pyrosequencing in Study 2 and sequencing in Study 3 [15]. To maintain consistency in the analysis of mixed infections, samples from Study 2 were analysed by pyrosequencing (*pfmdr1* D1246Y, *pfmdr1* Y184F), together with a dilution series of mixed DNA in different proportions from the clones 7G8 (*pfmdr1* 1246Y, *pfmdr1* 184F carrier) and 3D7 (*pfmdr1* D1246, *pfmdr1* Y184 carrier). Samples with more than 10% of each allele at a particular locus were defined as mixed infections. The cut off values were used to re-assess the data from Study 1 [10] to ensure consistency.

Sequencing was done by Macrogen Inc (Seoul, Korea), Sequencher™ software version 4.6 (Gene Codes Corporation, Ann Arbor, MI, USA) was used to analyse the sequences with 3D7 as the *pfmdr1* reference sequence (PFE1150w, Gene ID 813045 at NCBI RefSeq, National Center for Biotechnology Information Reference Sequence). The PCR success rates for *pfmdr1* N86Y, Y184F, D1246Y and *pfcrt* K76T were 97% (752/777), 96% (749/777), 95% (740/777) and 96% (749/777), respectively.

### *pfmdr1* copy number variation

In Study 1, 2, 3 and partly Study 4, *pfmdr1* copy number was assessed using Taqman^®^ based real-time PCR (ABI Prism^®^ 7000) [16]. *β-tubulin* was used as one copy endogenous control. The clones 3D7, K1, D10, all with one copy of *pfmdr1*, were used as calibrators. Dd2 and FCB were used as multi-copy controls. All samples were run in triplicates. PCR success rates were 84% (89/106), 96% (48/50), 81% (208/258) and 90% (45/50) for Studies 1, 2, 3 and 4, respectively.

### Ethical considerations

Before enrolment, written informed consent was obtained from parents/legal guardians of the children. The studies were approved by the National Institute for Medical Research, Tanzania and Karolinska Institutet Ethical Review Board or the Regional Ethics Committee, Stockholm, Sweden. Studies 2 and 3 were registered with identifier NCT00336375[17] and ISRCTN69189899[18], respectively.

### Statistical analysis

For prevalence analysis of individual SNPs, mixed infections (both alleles present at a particular locus) were analysed together with the polymorphism not associated with lumefantrine tolerance/resistance, i.e., *pfmdr1* 86Y, Y184, 1246Y and *pfcrt* 76 T. For haplotype analysis, minority haplotypes (≤ 5%) and infections that were mixed at two or more *loci* were excluded. Infections that were mixed at only one locus were analysed as having both haplotypes. The combined *pfmdr1* and *pfcrt* haplotype was made based on the *pfmdr1* haplotypes.

Logistic regression with year included as a continuous covariate was used to estimate the yearly changes in prevalence. The presented odds ratios (OR) with corresponding 95% confidence intervals (CI) represent the relative change per year. The 2006 study, conducted in June, i.e., just prior to artemether-lumefantrine deployment, was used as baseline for trend analysis. Statistical significances were confirmed using non-parametric trend test. STATA v. 12 was used for all analysis, figures were made in SigmaPlot^®^ 11, p-values were estimated using bootstrapping (100 repeats) and a p-value <0.05 was considered statistically significant.

## Results

### Description of study population

A total of 777 patients were included in the analysis. The details of the study population are presented in Table 1. The blood slide positivity rate for Study 5 was lower (17%) compared with the mean all other studies combined (53%).

### Temporal trends in the prevalence of SNPs in *pfmdr1* and *pfcrt* SNPs

**Figure 1:**
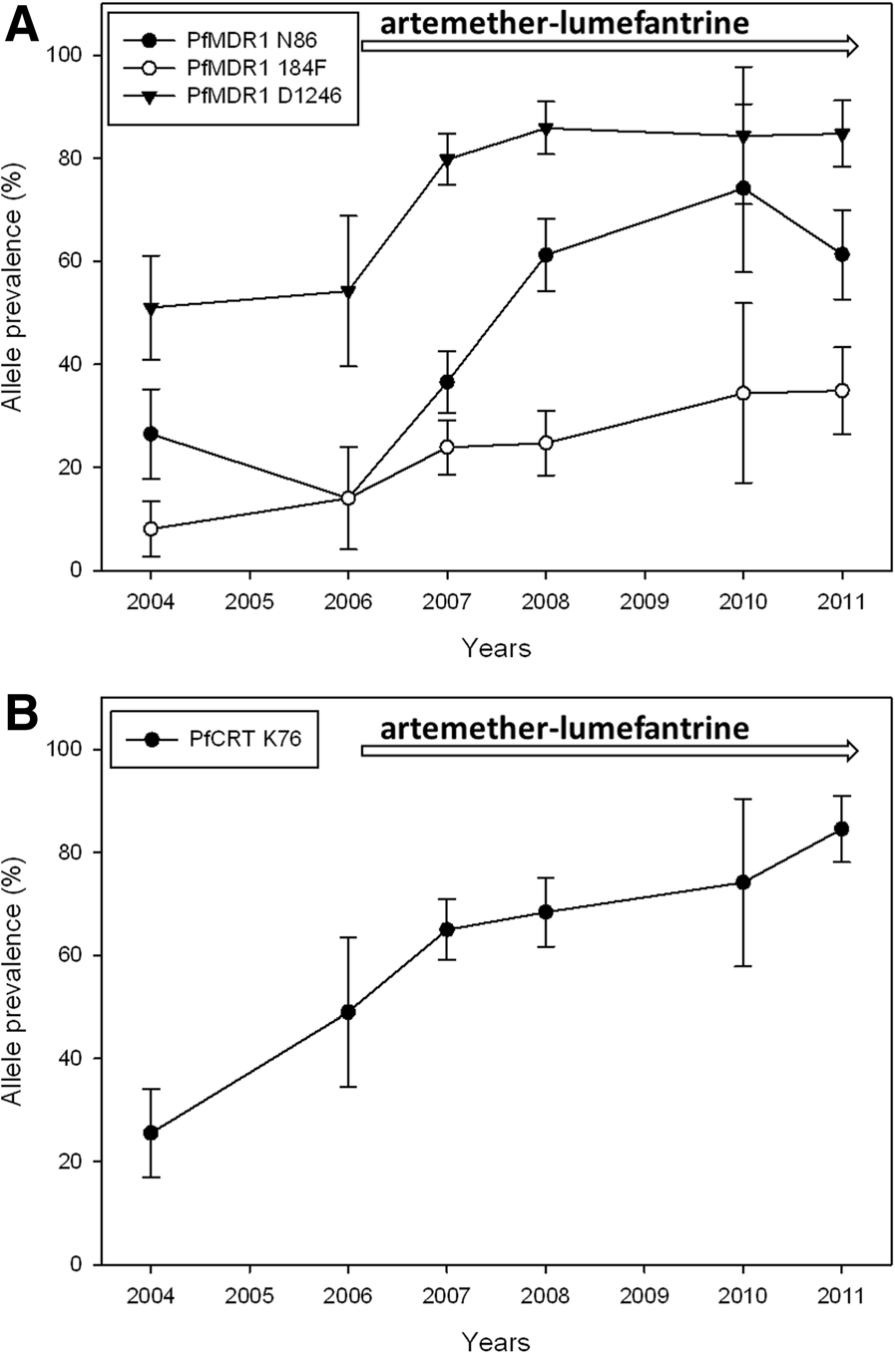
**Temporal trends of PfMDR1 N86Y, Y184F, D1246Y and PfCRT K76T.** Temporal trends (means and 95% confidence intervals) of **A**) PfMDR1 N86, 184F, D1246 and **B**) PfCRT K76 during 2004–2011 in Fukayosi village, Bagamoyo District, Tanzania. Mixed infections (both alleles present at a particular locus) were analysed together with the polymorphism not associated with lumefantrine tolerance/resistance, i.e., PfMDR1 86Y, Y184, 1246Y and PfCRT 76 T. The arrow indicates when artemether-lumefantrine has been first-line treatment.

There was a statistically significant increase of *pfmdr1* N86, 184F and D1246 over the time period 2006 to 2011 from 14% to 61% (yearly OR = 1.38 [95% CI 1.25-1.52] p < 0.0001), 14% to 35% (OR = 1.17 [95% CI 1.07-1.30] p = 0.001) and 54% to 85% (OR = 1.21 [95% CI 1.03-1.42] p = 0.016), respectively (Figure 1, Table 2). No significant yearly change was observed between 2004 and 2006. During the same time period there was a significant increase of *pfcrt* K76 from 49% pre-AL to 85% (OR = 1.33 [95% CI 1.17-1.51] p < 0.0001). However, a significant increase of *pfcrt* K76 was observed already between 2004–2006, from 26% to 49% (OR = 1.68 [95% CI 1.17-2.40] p = 0.005).

**Table 2:** **Prevalences of *pfmdr1 *** **and *pfcrt *** **SNPs in Fukayosi village, Bagamoyo District, Tanzania, from 2004-2011**

**Gene**	**Mutation**	**Study 1**		**Study 2**		**Study 3**		**Study 4**		**Study 5**		**Study 6**	
		**2004**		**2006**		**2007**		**2008**		**2010**		**2011**	
		n=	(%)	n=	(%)	n=	(%)	n=	(%)	n=	(%)	n=	(%)
*pfmdr1*	**N86**	27	26.5	7	14	94	36.6	115	61.1	23	74.2	76	61.3
	**mix-86**	30	29.4	19	38	71	27.6	27	14.4	3	9.7	20	16.1
	**86Y**	45	44.1	24	48	92	35.8	46	24.5	5	16.1	28	22.6
	**184F**	8	8	7	14	61	23.9	46	24.7	10	32.3	44	34.9
	**mix-184**	17	17	6	12	30	11.8	23	12.4	8	25.8	15	11.9
	**Y184**	75	75	37	74	164	64.3	117	62.9	13	41.9	67	53.2
	**D1246**	50	51	26	54.2	202	79.8	158	85.9	27	84.4	106	84.8
	**mix-1246**	24	24.5	14	29.2	20	7.9	15	8.1	4	12.5	14	11.2
	**1246Y**	24	24.5	8	16.7	31	12.3	11	6	1	3.1	5	4
*pfcrt*	**K76**	26	25.5	24	49	167	65	128	68.4	23	74.2	104	84.6
	**mix-76**	23	22.5	11	22.5	43	16.7	28	15	2	6.5	5	4.1
	**76T**	53	52	14	28.6	47	18.3	31	16.6	6	19.4	14	11.4

N = asparagine, Y = tyrosine, F = phenylalanine, D = aspartic acid, mix = the presence of both alleles at one *loci.*

### *pfmdr1* haplotypes

**Figure 2:**
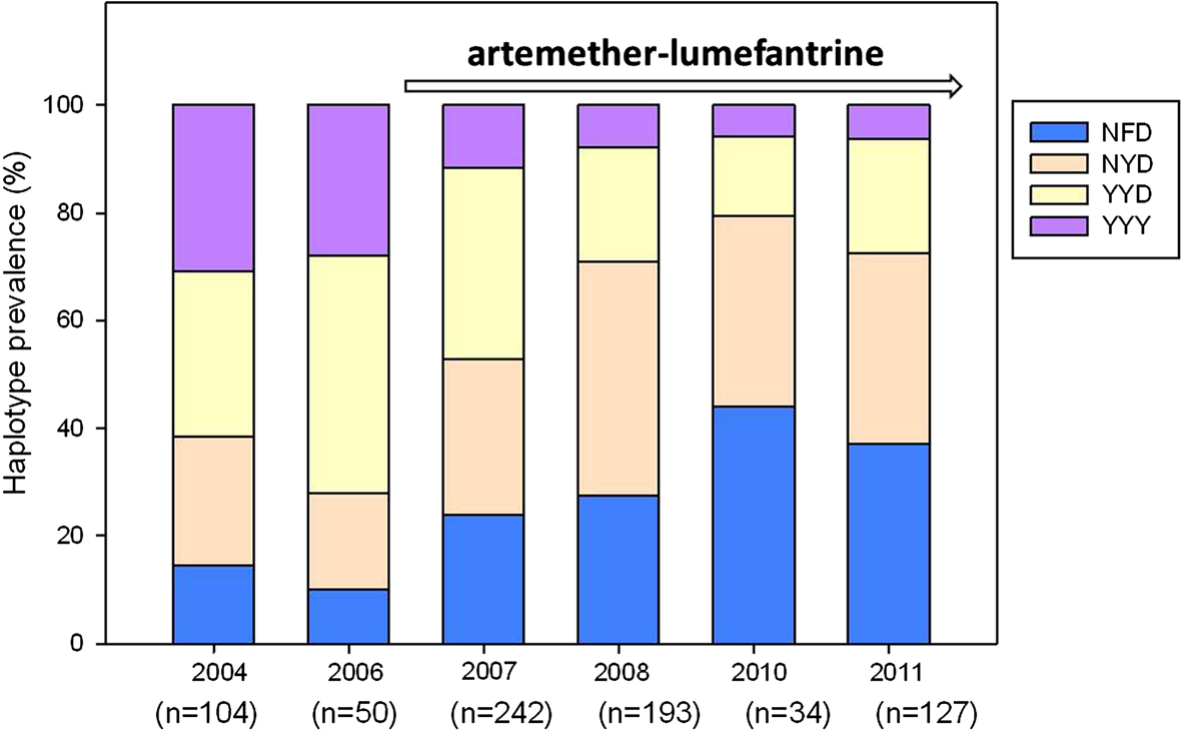
**Temporal trends of PfMDR1 haplotypes.** Prevalences of PfMDR1 haplotypes at codon N86Y, Y184F, D1246Y in Fukayosi village, Bagamoyo District, Tanzania, from 2004–2011. Minority haplotypes (≤ 5%) and infections that were mixed (both alleles present at a particular locus) at 2 two or more loci were excluded. Infections that were mixed at only one locus were analysed as having both haplotypes. The arrow indicates when artemether-lumefantrine has been first-line treatment for uncomplicated malaria.

The prevalences of *pfmdr1* haplotypes at codon N86Y, Y184F, D1246Y during 2004–2011 are presented in Figure 2. There was a statistically significant increase of NFD haplotype between 2006–2011 from 10% to 37% (OR = 1.25 [95% CI 1.12-1.39] p < 0.0001). During the same period the NYD showed a trend of increase from 18% to 35% (OR = 1.10 [95% CI 0.98-1.23] p = 0.098). The YYD and YYY haplotypes decreased significantly 2006 to 2011 from 44% to 21% (OR = 0.79 [95% CI 0.68-0.90] p < 0.0001) and 31% to 6% (OR = 0.73 [95% CI 0.56-0.98] p = 0.018), respectively.

### The combined *pfmdr1* and *pfcrt* haplotypes

There was a statistically significant increase of the NFD + K haplotype from 12% to 36% (OR 1.27 [95% CI 1.12-1.45] p < 0.0001) between 2006–2011. During the same period the NYD + K haplotype increased from 7% to 28% (OR 1.10 [95% CI 0.97-1.25] p = 0.127). Between 2006 and 2011 there was a significant decrease of the YYD + K and YYD + T from 29% to 20% (OR 0.86 [95% CI 0.74-1.00] p = 0.041) and 17% to 0% (OR 0.59 [95% CI 0.41-0.83] p = 0.003), respectively. During the same time period there was a decrease in prevalence of YYY + T from 19% to 2% (OR 0.63 [95% CI 0.39-1.00] p = 0.05). This decrease was statistically significant by non-parametric trend test (p = 0.003) (Table 3).

**Table 3:** **Prevalences of the combined *pfmdr1 *** **and *pfcrt *** **haplotypes in Fukayosi village, Bagamoyo district, Tanzania, from 2006-2011**

**Haplotype**	**2006**	**2007**	**2008**	**2010**	**2011**	**Yearly OR**	**95% CI**	**p-value logistic regression**	**p-value non-parametric trend test**
**NFD + K**	12% (5/42)	20% (46/234)	21% (40/188)	37% (13/35)	36% (43/120)	1.27	1.12-1.45	0.000	0.000
**NYD + K**	7% (3/42)	23% (53/234)	36% (68/188)	29% (10/35)	28% (34/120)	1.10	0.97-1.25	0.127	0.093
**YYD + K**	29% (12/42)	27% (64/234)	14% (27/188)	9% (3/35)	20% (24/120)	0.86	0.74-1.00	0.041	0.022
**YYY + K**	10% (4/42)	7% (16/234)	4% (7/188)	0% (0/35)	4% (5/120)	0.79	0.59-1.06	0.110	0.079
**NFD + T**	0% (0/42)	5% (12/234)	5% (10/188)	9% (3/35)	3% (3/120)	0.96	0.76-1.21	0.732	0.746
**NYD + T**	7% (3/42)	5% (12/234)	8% (15/188)	6% (2/35)	8% (9/120)	1.05	0.87-1.26	0.624	0.640
**YYD + T**	17% (7/42)	7% (17/234)	7% (14/188)	6% (2/35)	0% (0/120)	0.59	0.41-0.83	0.003	0.000
**YYY + T**	19% (8/42)	6% (14/234)	4% (7/188)	6% (2/35)	2% (2/120)	0.63	0.39-1.00	0.050	0.003

The haplotypes refers to amino acid positions *pfmdr1* N86Y, Y184F, D1246Y and *pfcrt* K76T. Yearly OR is the Odds Ratio for logistic regression 2006 until 2011 with year included as a continuous covariate. The OR can thus be interpreted as the relative change per year. N = asparagine, Y = tyrosine, F = phenylalanine, D = aspartic acid, K = lysine, T = threonine.

### *pfmdr1* copy number variation

All 390 successfully analysed samples had one copy of the *pfmdr1* gene.

## Discussion

This study provides evidence for a continuous selection of molecular markers associated with artemether-lumefantrine tolerance/resistance in the local *P. falciparum* population in Fukayosi village, Bagamoyo district, Tanzania, occurring after the introduction of this ACT as first-line treatment for uncomplicated malaria in 2006.

The results support previous observations of *pfmdr1* N86 selection in Gabon, Kenya and Mozambique [19-21] as well as selection of both *pfmdr1* N86 and 184F in Korogwe, Tanzania [22], and the *pfmdr1* N86, 184F, D1246 haplotype in Mozambique [23] following wide scale deployment of artemether-lumefantrine. However, the present report adds substantially to the evidence base being more comprehensive both with regards to number of patients and genetic markers analysed, and importantly with a longer duration of follow-up.

Interestingly the selection of *pfcrt* K76 started already prior to the introduction of artemether-lumefantrine in Bagamoyo district. This probably represents an effect of the withdrawal of chloroquine as first-line treatment in 2001, consistent with observations from Malawi where withdrawal of chloroquine resulted in a fast re-expansion of a diverse chloroquine-susceptible *pfcrt* K76 population [24]. Thus, the herein observed increase in *pfcrt* K76 may not necessarily only be due to the introduction of artemether-lumefantrine, but could also, at least partly, be explained by the withdrawal of chloroquine and/or other factors, such as parasite fitness and transmission intensity [24-26]. Conversely, no selection of *pfmdr1* N86, 184F, D1246 occurred prior to introduction of artemether-lumefantrine in the study area. The selection of these SNPs seen after 2006 is therefore unlikely driven by the withdrawal of chloroquine.

There are evidences that exposure of artemether-lumefantrine is the main contributor behind the observed selection of *pfmdr1* N86, 184F, D1246 SNPs and that it plays a role also for selection of *pfcrt* K76. These evidences include the previously reported specific lumefantrine-driven selection among re-infections during follow up after artemether-lumefantrine treatment [9,10], *in vitro* findings [27] and a recent study conducted in Tanzania, which shows that the selection of N86, 184F and D1246 after artemether-lumefantrine treatment *in vivo* is significantly associated with the ability to withstand higher lumefantrine concentrations [15]. In this context it is also worth noting that there are studies suggesting that both the artemisinin-derivatives and lumefantrine select for the same molecular markers [28,29]. This, together with the recent evidence from Southeast Asia that *P. falciparum* is able to develop artemisinin tolerance/resistance, is of particular concern as it could result in an additive or even synergistic selection of molecular markers of anti-malarial drug resistance in the parasite population.

It is of note that the blood slide positivity rate in Study 5 was lower (17%) compared with the mean for all other studies combined (53%). This may be due to that Study 5 was conducted during October-January, when the malaria transmission is relatively low. However, this did not appear to have influenced the SNP prevalences. Furthermore, it is important to underline that clinical efficacy of artemether-lumefantrine remained high in the study area with PCR-corrected cure rate >95% in 2007 [7]. Nevertheless, in an era when the number of malaria patients is slowly declining, standard *in vivo* trials are increasingly difficult and costly to conduct. In this context, molecular surveillance may play an important role to detect selection of genetic markers associated with ACT tolerance/resistance in the local *P. falciparum* population over time.

## Conclusions

Increased prevalence of *pfmdr1* N86, 184F, D1246 and *pfcrt* K76 was observed in the parasite population after deployment of artemether-lumefantrine as first-line treatment for uncomplicated malaria. The *pfmdr1* haplotype NFD increased significantly at the cost of YYY and YYD. The temporal selection of molecular markers associated with artemether-lumefantrine tolerance/resistance may represent an early warning sign of impaired future drug efficacy. This calls for stringent surveillance of artemether-lumefantrine efficacy in Tanzania and emphasizes the importance of molecular surveillance as a complement to standard *in vivo* trials.

## Competing interests

The authors declare that they have no competing interests.

## Authors’ contributions

MM carried out the molecular analysis, participated in the design, coordinated the work and drafted the manuscript. BN and ZP participated in the planning and conduct of the field trials. EL and AH, participated in extraction and genotyping. IJ performed copy number variation analysis. MP assisted in the statistical analysis. PEF, JPG and AB participated in the design of the study and interpretation of results. AM participated in the planning and conduct of the field trials, designed of the study and interpretation of results. All authors critically revised the manuscript and approved the final version of the manuscript.
